# Usefulness of Skin Autofluorescence as a Biomarker of Acute Oxidative Stress in Young Male Japanese Long-Distance Runners: A Cross-Sectional Study

**DOI:** 10.3390/sports10110180

**Published:** 2022-11-16

**Authors:** Rei Fujiwara, Natsume Anzai, Motoyasu Ishikawa, Atsuhiko Takahashi

**Affiliations:** 1 Department of Food and Nutrition, Junior College, Nihon University, 2-31-145 Bunkyo-Cho, Mishima 411-8555, Shizuoka, Japan; 2 Department of Philosophy, Division of Humanities, Graduate School of Humanities, Osaka University, 1-5 Machikaneyama-Cho, Toyonaka 560-8532, Osaka, Japan

**Keywords:** advanced glycation end products, skin autofluorescence, pentosidine, d-ROMs, BAP, BAP/d-ROMs ratio, athletes, runner

## Abstract

Chronic oxidative stress in long-distance runners adversely affects conditioning. It is important to objectively assess and monitor oxidative stress, but measuring oxidative stress can be invasive or require skill to measure. Therefore, this study aimed to verify whether skin autofluorescence (SAF), a non-invasive, rapid, and easily calculable metric for calculating advanced glycation end products (AGEs), is useful as an oxidative stress biomarker. The subjects were 50 young Japanese male long-distance runners (aged 20.2 ± 1.2 years); 35 average-sized male university students (aged 19.8 ± 1.1 years) served as controls. The interactions and relationships between SAF and plasma pentosidine and oxidative stress markers (reactive oxygen metabolite-derived compounds [d-ROMs], biological antioxidant potential [BAP], and the BAP/d-ROMs ratio) in runners were examined, and SAF in the runners and controls was compared. The results suggest that plasma pentosidine in runners is associated with oxidative stress markers and that it can assess oxidative stress. However, as SAF was not associated with oxidative stress markers, it was not validated as one. In future, clarifying the factors affecting SAF may also clarify the relationship between SAF, plasma pentosidine, and oxidative stress markers.

## 1. Introduction

Long-distance runners’ prolonged and intense exercise induces oxidative stress when the body’s defence mechanisms are overwhelmed by the formation of reactive oxygen species (ROS), a collective term for oxygen molecules derived from highly reactive oxygen molecules (O_2_) and others [1]. Oxidative stress has been shown to adversely affect skeletal muscle fatigue and immune function [2,3]. Their chronicity in long-distance runners may lead to adverse effects on conditioning. Therefore, quantification, objective assessment, and monitoring of oxidative stress are important to minimise these risks. Although several biomarkers exist to assess exercise-induced oxidative stress in athletes, measurement of these markers is invasive because it requires blood sampling, or because of the unstable nature of ROS, the several markers require specialized technology to measure [4]. Assessment of oxidative stress should be non-invasive and simple for applications, including the early detection of the risk of sports injuries and overtraining.

High oxidative stress in vivo promotes the formation of advanced glycation end products (AGEs) [5]. Pentosidine, a major AGE, is formed by non-enzymatic reactions and oxidation processes of arginine residues and lysine groups, and unlike the unstable ROS, it is a product with a stable structure and exhibits fluorescence and cross-linking [6]. Because of its properties, pentosidine is useful as a marker to assess and predict the onset and progression of diseases associated with oxidative stress [7,8]. AGEs with fluorescent functions, including pentosidine, can be estimated noninvasively, quickly, and simply as skin autofluorescence (SAF) using the AGE Reader [9]. SAF has been reported to be associated with a number of diseases affected by chronic glycation and oxidative stress. It is a useful predictive biomarker for the evaluation of peripheral artery disease (PAD) and cardiovascular disease mortality in patients with renal failure and diabetes [10,11,12,13,14]. Although SAF measured with the AGE Reader is thought to reflect AGEs in skin tissue that have accumulated in long-lived proteins, usually over several years, transient changes have been observed in SAF as well as circulating AGEs under assumed conditions of rapid oxidative stress [15].

In summary, SAF has the potential to be used to noninvasively assess acute oxidative stress, but to our knowledge, no reports have been investigated in athletes with a focus on this metric. Therefore, this study was designed to examine the interactions and relationships between SAF and plasma pentosidine and oxidative stress-related markers, to test whether SAF is a valid assessment index to estimate oxidative stress in long-distance runners. Study 1 used controls as a point of comparison to obtain basic data on SAF in long-distance runners. Study 2 examined the correlation between SAF, plasma pentosidine, and oxidation-related markers. Study 3 examined whether SAF evaluated plasma pentosidine in long-distance runners. The hypothesis of this study is as follows: SAF in runners reflects plasma pentosidine concentrations and is associated with oxidative stress biomarkers.

## 2. Materials and Methods

### 2.1. Subjects

The study had a cross-sectional design. Participation in the study was by invitation. All surveys were conducted in August–September 2020. The participants in this study consisted of 52 long-distance runners from the distance division of the men’s track and field athletes belonging to the University group and 39 male college students as the control group. All study participants were free of chronic diseases (for example, diabetes, atopy, and other skin diseases). A reliable measure of SAF is possible in subjects with skin reflectance greater than 6% from I to IV of the Fitzpatrick skin typing [16,17], and no subjects with type V or higher were included in this study.

The final analysis included 50 and 35 participants in the runners and control groups, respectively. Two persons who abstained from blood collection were excluded from the analysis of runners. In the case of the control group, three individuals in whom SAF could not be determined, as well as one smoker, were excluded. Smoking was used as an exclusion criterion because tobacco increases the concentration of AGEs in the body [18].

The runners’ athletic status was at a comparatively elite level of competitiveness among universities, practicing twice a day, six times a week. The controls confirmed prior to conducting the study that they were not athletes and were not involved in any club activities that included physical activity. Runners’ bodies and SAF levels were measured at the subjects’ dormitory (Tokyo, Japan), and blood samples were taken at a general hospital (Tokyo, Japan). The bodies of controls and SAF were measured in a classroom on the university campus (Shizuoka, Japan).

This study was approved by the Ethics Review Committee of the College of International Relations of Nihon University (2020-001) on 6 August 2020, and all procedures were performed in compliance with the Declaration of Helsinki. All subjects were informed orally and in writing about the purpose and methods of this study in advance, and their written consent to participate in the study was obtained after they fully understood the content of the study.

### 2.2. Anthropometry

Height was measured using a height meter with an accuracy of 0.1 cm. Runners’ weight were measured using InBody720 (Inbody Japan Co., Tokyo, Japan). Controls’ body weights were measured using a multi-frequency body composition analyser, MC-190 (Tanita Corp., Tokyo, Japan). Body mass index (BMI) was calculated by dividing weight (kg) by height squared (m^2^).

### 2.3. SAF

SAF was measured using the AGE Reader (AGE Reader, Diagnoptics Technologies B.V., Groningen, The Netherlands; Japanese import agent, Serista Corporation). The instrument irradiates the skin with an excitation wavelength (300–420 nm), measures the fluorescence wavelength (420–600 nm) emitted from the skin, and automatically expresses the integral value of the fluorescence intensity as SAF value (unit: AU). Higher values indicate higher amounts of AGEs. Measurements were taken in a sitting position, and the measurement site was the inner forearm (5–10 cm below the elbow). Previous studies have established the validity and reliability of this measurement site for measuring SAF values in Japanese people [19]. A location free of scars, moles, or bruises was selected, and adjacent areas were measured three times, shifting the location slightly, and the average value was used as the representative value. Since products such as skin lotions and sunscreens affect the measurement results [20], we ensured that the subjects did not use them. As a precaution, the measurement site was disinfected with an alcohol wipe and dried well before measurement. 

### 2.4. Biochemical Measurements

Blood samples were collected only from runners. Blood samples were drawn from the elbow vein on the same day as body composition and SAF measurements. In terms of AGE concentration, plasma pentosidine levels (FSK PEN ELISA kit; Fushimi Pharmaceutical Co., Marugame, Japan) were tested using enzyme-linked immunosorbent assay (ELISA) kits. The FSK PEN ELISA kit has been found to correlate well with values measured by high-performance liquid chromatography (*r* = 0.936) [21]. In addition, creatine-kinase (CK) and lactate-dehydrogenase (LDH), indicators of muscle damage, were included in the survey as secondary outcomes. Their analysis was outsourced to a laboratory (SRL, Inc., Tokyo, Japan).

Since the oxidative stress of a living body is determined by the equilibrium between oxidation and anti-oxidation, it is necessary to evaluate the phenomenon as oxidative balance by measuring oxidation alongside multiple other biomarkers. Reactive oxygen metabolite-derived compounds (d-ROMs) and biological antioxidant potential (BAP) can measure and evaluate oxidative stress and antioxidant capacity, respectively [22,23,24]. By combining them, the ratio of BAP/d-ROMs can be calculated, which indicates the equilibrium and balance between oxidative stress and antioxidant capacity. The collected blood was immediately centrifuged at 3000 rpm for 10 min and stored in a freezer until assay. Each serum sample was analysed using a free radical analyser (FREE Carrio Duo, Diacron International SRL, Grosseto, Italy). The free radical analyser is an instrument equipped with a photometer, which automatically calculates d-ROMs and BAP values after inserting a cuvette into the measuring cell. The d-ROMs test can comprehensively evaluate oxidative stress in vivo by measuring the concentration of plasma hydroxyperoxide, produced by reactive oxygen species and free radicals in the body, using a colour reaction. Its validity has been proven by its correlation with free radical values using the electron spin resonance method [24].

For the assay procedure, 20 µL of serum sample was placed in a cuvette containing pH 4.8 buffer and gently inverted and stirred. Next, 20 µL of colourless chromogen (N, N-diethyl-paraphenylenediamine) was added to the cuvette, gently inverted, and stirred again, and the absorbance at 505 nm was measured. The principle of this measurement is that when blood is diluted with an acidic buffer solution of pH 4.8, Fe^2+^ and Fe^3+^ are separated from blood proteins and then act as catalysts to decompose the hydroperoxide in the blood into alkoxyl and peroxy radicals. The d-ROMs test value is calculated by calculating the change in absorbance of the coloured radical cations when adding a colourless colouring solution. The unit of measure is U.CARR, where 1 U.CARR corresponds to 0.08 mg/dL of hydrogen peroxide. The BAP test can evaluate antioxidant capacity by measuring the ability of an oxidant to give electrons to reactive oxygen species/free radicals and stop oxidative reactions. 

For the BAP test procedure, each serum sample (10 µL) was placed in a cuvette containing BAP colouring solution (50 µL) and inverted and stirred. The cuvettes were then placed in a thermostat for 5 min and then set in a photometer to measure absorbance at 505 nm. The principle of this measurement is that the trivalent iron salt FeCl_3_ in the BAP colouring solution turns red as a function of trivalent iron Fe^3+^ ions when dissolved in a colourless solution containing a certain thiocyanate derivative. However, when added, plasma is reduced to divalent iron Fe^2+^ ions by the action of antioxidants in the plasma and is decolourized. The BAP test value is calculated by measuring the amount of change in its colour. The coefficients of variation in previous studies ranged from 0.2 to 2.1% for d-ROM and from 0.1 to 1.1% for BAP [25].

### 2.5. Self-Administered Questionnaire Survey

For the survey on mileage in runners, respondents were asked to answer, “Distance in km” to the question “Please tell us your weekly mileage (from the previous day to the past week)”.

The survey on exercise frequency given to the controls asked the question, “Do you currently exercise regularly?” and asked them to answer in terms of, “Type of activity, time/day, frequency/week.” If they did not exercise, they were asked to answer “none”.

### 2.6. Statistical Analysis

Each statistical method was analysed using SPSS, version 28 (IBM, Japan Inc., Tokyo, Japan). The significance level was set at 5% with a two-tailed test. The normality of each variable was evaluated using the Shapiro–Wilk test. Results are expressed as means ± standard deviations or the median values (25–75% IQR). All analyses used normal or non-normal based test methods. Comparisons between two independent groups were made using either the unpaired t-test or the Mann–Whitney’s U test. Spearman’s rank correlation coefficient was used for correlation. In addition, although SAF is usually positively correlated with age, we did not adjust for age as a confounding factor because of the very narrow age range of the subjects in this study. 

## 3. Results

### 3.1. Subjects’ Characteristics (Study 1)

The characteristics of the subjects (runners, n = 50; controls, n = 35) are shown in Table 1. The mean age of the runners was 20.3 ± 1.2 years, and BMI was 19.1 ± 1.1 kg/m^2^. SAF, an indicator of AGEs, was 1.20 (1.0–1.3) AU, and plasma pentosidine was 47.3 ± 10.3 ng/mL. The d-ROMs test, a measure of exercise-related fatigue, scored 274 ± 42 U.CARR BAP test was measured to be 2143 (2034–2291) µmol/L. The BAP/d-ROMs ratio was 8.2 (7.4–9.9). CK, an indicator of muscle fatigue, was 406.3 ± 310.5 U/L, and LDH was 213.5 ± 39.6 U/L. BMI in the controls was within the range of the average BMI of Japanese 15–29-year-olds (21.1 ± 3.6–22.9 ± 4.1 kg/m^2^) [26], confirming that the controls were indeed a group of average-sized Japanese men.

A comparison of the survey items between runners and controls showed that runners had significantly lower weight, BMI, and SAF than controls, while age and height were not significantly different (all *p* > 0.05).

### 3.2. Correlation between SAF and Anthropometric Values (Study 1)

Correlations between SAF and anthropometric values in runners and controls were examined (Table 2). The results showed that SAF in runners and controls were not associated with anthropometric values (age, height, weight, and BMI) (all *p* > 0.05).

### 3.3. Correlation between SAF and Plasma Pentosidine and Markers of Oxidative Stress and Muscle Damage (Study 2)

**Figure 1:**
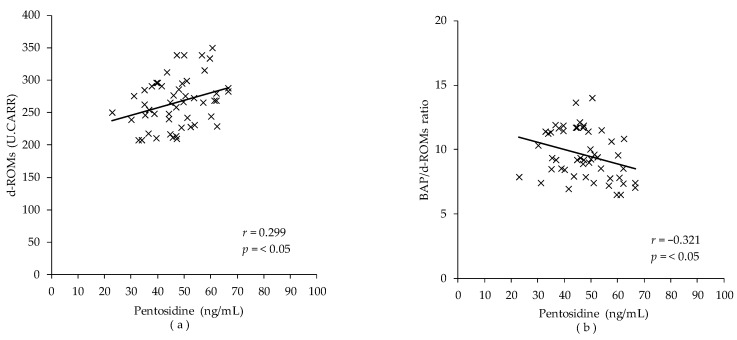
Correlation between plasma pentosidine and markers of oxidative stress in runners (n = 50). The correlation was calculated using the Spearman correlation analysis. (**a**) Correlation between plasma pentosidine and diacron-reactive oxygen metabolites (d-ROMs); (**b**) correlation between plasma pentosidine and BAP/d-ROMs ratio.

Correlations between SAF and plasma pentosidine and oxidation-related markers in runners were examined (Table 3) (Figure 1). The results showed that plasma pentosidine was positively correlated with d-ROMs (*r* = 0.299, *p* = 0.035) and negatively correlated with the BAP/d-ROMs ratio (*r* = −0.321, *p* = 0.023). SAF was not associated with any markers of oxidative stress and muscle damage (all *p* > 0.05).

### 3.4. Correlation between SAF and Plasma Pentosidine in Runners (Study 3)

**Figure 2:**
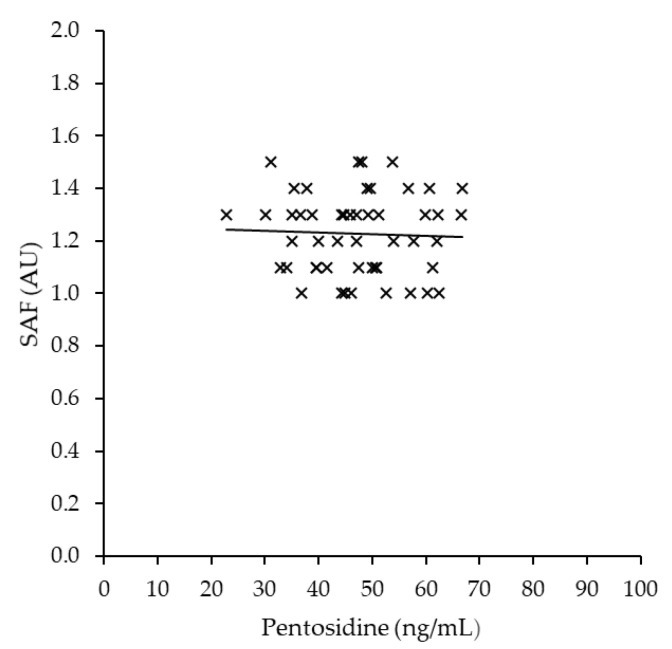
Correlation between SAF and plasma pentosidine in runners (n = 50). The correlation was calculated using the Spearman correlation analysis.

When the correlation between SAF and plasma pentosidine in runners was examined, there was no significant difference (*r* = −0.023, *p* = 0.872; Figure 2).

## 4. Discussion

This study aimed to test whether SAF is a valid assessment index to estimate acute oxidative stress in young Japanese long-distance runners and reported the interrelationship between SAF and plasma pentosidine and oxidative stress-related markers. This is the first report of its kind. Plasma pentosidine was positively correlated with d-ROMs and negatively correlated with BAP/d-ROMs ratio, suggesting that plasma pentosidine concentration in long-distance runners is valuable in evaluating oxidative stress and oxidative stress balance. However, SAF was not associated with plasma pentosidine and oxidative stress-related markers, and the usefulness of SAF as an indicator for assessing oxidative stress was not confirmed in this study. 

The plasma pentosidine concentration of runners in this study was 47.3 ± 10.3 ng/mL (Table 2), which is higher than the reference value. The reference value, according to the manufacturer of the ELISA-based pentosidine test kit used in this study, is 0.00915–0.0431 µg/mL (9.2–43.1 ng/mL) [27]. Although regular exercise is believed to reduce AGE concentrations because it suppresses the formation of reactive oxygen species [28], acute and high-intensity exercise may cause an overproduction of reactive oxygen species and a progressive increase in blood AGE concentrations. This paradox could be solved through this study; it is desirable to accumulate research reports focusing on the effects of oxidative stress due to high-intensity exercise on AGE accumulation.

Study 2 showed that plasma pentosidine concentrations were not only positively correlated with d-ROMs, the total oxidant capacity (*r* = 0.299, *p* = 0.035), but also negatively correlated with BAP/d-ROMs ratio, an index of antioxidant potential (*r* = −0.321, *p* = 0.023; Table 3). Under oxidative stress, the production of sugar-derived carbonyl compounds, precursors of pentosidine, is enhanced, and pentosidine levels increase [29]. In addition, AGEs bind ligands to RAGE and activate NAPDH oxidase, which enhances ROS formation [30,31]. These relationships suggest that plasma pentosidine level is a marker that can assess oxidative stress status in long-distance runners. The reason for the weak correlation between pentosidine and oxidation-related markers could be due to the difference in the products they measure. d-ROMs measure peroxide in the serum, representing a more acute oxidative stress. Pentosidine measures downstream products of oxidation in proteins that are being turned over, representing a mid-term oxidative stress level.

On the other hand, SAF was not associated with oxidative stress markers and plasma pentosidine (Table 3; Figure 2). It has been suggested that SAF temporarily altered under acute oxidative stress, such as after surgery [15]. Additionally, the possibility that SAF is able to measure the increase and decrease in blood AGEs levels caused by oxidative stress has been pointed out in a review article [32]. However, in this study, SAF, which indicates long-term oxidative stress, was not associated with d-ROM, which is indicative of acute oxidative stress. Therefore, it can be said that this study makes it clearer that SAF does not indicate acute oxidative stress levels. This is consistent with previous studies reporting that SAF failed to detect certain blood AGEs and was not consistent with variations in oxidative stress markers [33,34]. This may be due to the difference in metabolic turnover rates of AGEs in tissue and blood. The metabolic turnover rate of most proteins in the blood may be higher than long-lived proteins in tissues, such as collagen. It is also possible that fluorescent substances present in the skin affect SAF. Since SAF is calculated from the ratio of skin fluorescence to reflectance using light, it is influenced by several factors in the skin, including skin reflectance [35] and the presence of fluorescent substances in the skin [36,37]. In addition, melanin is a fluorescent substance and absorbs light in the ultraviolet and visible regions, and there has been concern that elevated melanin levels due to sun exposure may be a confounding factor for SAF [32]. Since the forearm is a site that does not tan throughout the year, risk factors due to sun exposure did not need to be considered in non-Athletes. However, because athletes training outdoors routinely have longer UV exposure times, the skin of athletes may be more affected by tan than non-Athletes [38]. The SAF for this study was measured in late August-early September. The Global Solar UV Index (UVI), an indicator of the level of solar ultraviolet radiation that comprehensively evaluates the degree of impact on the human body [39], was 7.4 in August [40], which was the highest value in Japan compared to other seasons; it was a time when there was an impact of ultraviolet radiation compared to other seasons.

Study 1 also showed that SAF in runners was significantly lower than in controls (*p* = 0.011). The results might have also been influenced by the SAF-related factors mentioned above. Runners had lower SAF values than controls, possibly due to skin pigmentation caused by tan and the absorption of excitation light necessary to excite fluorescent AGEs by the fluorescent substances (such as melanin) in the skin, which were elevated by tan.

Another reason could be that the beneficial effects of exercise might have worked to reduce glycation, and the amount of AGEs in the body was lower than that in controls of the same age group. The exercise frequency for the controls in this study was 0.8 ± 1.4 times/week, and the exercise duration was 32.6 ± 63.6 min/week. These did not meet the WHO recommendation of at least twice a week, 150–300 min of moderate-intensity exercise or 75–300 min of high-intensity exercise [41]. Thus, the controls in this study were a relatively inactive population. Regarding the relationship between SAF and the amount of physical activity, several studies have reported that an increase in physical activity leads to a decrease in SAF [42,43]. However, there exist reports of no association between physical activity and SAF [44]. Furthermore, a previous study examining SAF in runners reported that SAF in runners and untrained controls was not significantly different [42,43]. Thus, the average of SAF in runners and the effect of exercise on SAF remain controversial. Based on the above, we believe that a longitudinal examination of SAF, considering the influencing factors, will clarify basic data on SAF in runners and the relationship between SAF and plasma AGE concentration.

This study had several limitations. First, because this was a cross-sectional study, it was not possible to discuss the causal relationship between SAF and changes in oxidative stress markers, nor SAF and plasma pentosidine. Therefore, it is necessary to examine these longitudinally. In addition, this study did not validate the controls’ oxidative stress markers and plasma pentosidine. All of the participants had practiced with low intensity the day before; thus, more than 12 h had passed since their last exercise. However, because the intensity of the practice and the distance travelled varied from player to player, we have not standardized that part of the study. This may affect the oxidative stress values. It is possible that verifying these values in the controls could also clarify the nature of oxidative and glycative stress in runners and the relationship between them and SAF. In the present study, we examined the association of AGE concentration (plasma pentosidine) and oxidative stress markers (d-ROM, BAP, and BAP/d-ROM ratio) with SAF. Pentosidine was measured using the ELISA method. This method has been reported to involve some degree of uncertainty because several factors in human serum interfere with the AGE antibody response in ELISA [45]. The possible systematic errors caused by the aforementioned problems might have underestimated the effect of pentosidine. Although the oxidative stress-related indices were evaluated by d-ROM and BAP, the relationship between SAF and oxidative stress could have been examined in more detail by using multiple markers. d-ROM is not a marker that directly measures ROS or free radicals in vivo. It is a relatively new method to comprehensively evaluate the state of oxidative stress in the body by measuring the concentration of hydroperoxides in the blood, which are mainly caused by ROS and free radicals, using a colour reaction. In general, oxidation-related markers include direct evaluation of ROS and free radicals, indirect evaluation by measuring reaction products by ROS, and evaluation of 8-hydroxy-deoxyguanosine in urine as an indicator of deoxyribonucleic acid oxidation. Since there are many types of ROS and antioxidants, it is difficult to say that all oxidative stress and antioxidant capacity in vivo could be evaluated by measuring only one marker, as in this study. On the other hand, it is also true that it is difficult to identify all ROS and antioxidants even if multiple evaluation markers are examined identically; thus, care must be taken in interpreting the results. In addition, the effect of exercise on oxidative stress levels is not yet clear, and future studies should be conducted in a group with known variation in oxidative stress levels. Finally, this study did not examine tanning, skin colour, or other factors that affect SAF. The Fitzpatrick test of the subjects in this study is limited to III to IV. This is possibly because the classification method of the Fitzpatrick test is not suitable for determining skin color in the Japanese individuals. Therefore, in the future, skin color should be strictly measured and investigated using a colorimeter for sun-tanning in Japanese individuals. Future studies may clarify the relationship between this investigation and oxidative stress by simultaneously examining factors that may affect SAF.

Despite these limitations, the present study suggests that plasma pentosidine in long-distance runners is associated with oxidative stress markers and that it can assess oxidative stress. However, SAF was not associated with oxidative stress and plasma pentosidine and was not validated as an oxidative stress marker. In the future, clarifying the factors affecting SAF may clarify the relationship between SAF, plasma pentosidine, and oxidative stress.

## Figures and Tables

**Table 1:** Subject characteristics.

Characteristics	Runners (n = 50)	Controls (n = 35)	*p*-Value
Age, year (SD)	20.2 ± 1.2	19.8 ± 1.1	0.061
Height, cm (SD)	169.8 ± 5.0	170.3 ± 6.4	0.510
Weight, kg (SD)	55.3 ± 4.0	65.4 ± 3.1	<0.001
Body mass index, kg/m^2^ (SD)	19.1 ± 1.1	22.6 ± 1.0	<0.001
Skin autofluorescence, AU (IQR)	1.20 (1.0–1.3)	1.30 (1.2–1.4)	0.011
Pentosidine, ng/mL (SD)	47.3 ± 10.3	_	_
d-ROMs, U.CARR (SD)	274 ± 42	_	_
BAP, µmol/L (IQR)	2143 (2034–2291)	_	_
BAP/d-ROMs ratio (IQR)	8.2 (7.4–9.9)	_	
CK, U/L (SD)	406.3 ± 310.5	_	_
LDH, U/L (SD)	213.5 ± 39.6	_	_
Mileage, km/week (SD)	102.4 ± 52.0	_	_
Frequency of Exercise, times/week (IQR)	_	0.0 (0.0–1.0)	_
Exercise time, min/week (IQR)	_	0.0 (0.0–60)	_

Values are mean ± SD and medians [IQRs]. Between-group comparisons were performed using the Student’s *t*-test or Mann–Whitney U test. d-ROMs, diacron-reactive oxygen metabolites; BAP, biological antioxidant potential; CK, creatine kinase; LDH, lactate dehydrogenase.

**Table 2:** Correlation between SAF and anthropometric values.

	Skin Autofluorescence (AU)			
	Runners (n = 50)		Controls (n = 35)	
	*r*	*p*	*r*	*p*
Age, year	0.008	0.955	0.195	0.229
Height, cm	−0.128	0.387	−0.252	0.062
Weight, kg	−0.126	0.394	0.008	0.962
Body mass index, kg/m^2^	−0.025	0.868	0.121	0.471

Correlation coefficients (*r*) and *p* are calculated using the Spearman correlation analysis.

**Table 3:** Correlation of SAF and plasma pentosidine with markers of oxidative stress and muscle damage in runners (n = 50).

	Skin Autofluorescence (AU)		Pentosidine (ng/mL)	
	*r*	*p*	*r*	*p*
d-ROMs, U.CARR	0.124	0.391	0.299	0.035 *
BAP, μmol/L	−0.108	0.457	−0.134	0.352
BAP/d-ROMs ratio	−0.187	0.192	−0.321	0.023 *
CK, U/L	0.179	0.215	0.037	0.799
LDH, U/L	0.190	0.187	−0.084	0.562

Correlation coefficients (*r*) and *p* are calculated using the Spearman correlation analysis. d-ROMs, diacron-reactive oxygen metabolites; BAP, biological antioxidant potential; CK, creatine kinase; LDH, lactate dehydrogenase. * *p* < 0.05.

## Data Availability

The data presented in this study are available on request from the corresponding author. The data are not publicly available due to privacy reasons.
